# Diagnostics and treatment of diffuse intrinsic pontine glioma: where do we stand?

**DOI:** 10.1007/s11060-019-03287-9

**Published:** 2019-09-14

**Authors:** Fatma E. El-Khouly, Sophie E. M. Veldhuijzen van Zanten, Vicente Santa-Maria Lopez, N. Harry Hendrikse, Gertjan J. L. Kaspers, G. Loizos, David Sumerauer, Karsten Nysom, Kaie Pruunsild, Virve Pentikainen, Halldora K. Thorarinsdottir, Giedre Rutkauskiene, Victor Calvagna, Monika Drogosiewicz, Monica Dragomir, Ladislav Deak, Lidija Kitanovski, Andre O. von Bueren, Rejin Kebudi, Irene Slavc, Sandra Jacobs, Filip Jadrijevic-Cvrlje, Natacha Entz-Werle, Jacques Grill, Antonis Kattamis, Peter Hauser, Jane Pears, Veronica Biassoni, Maura Massimino, Enrique Lopez Aguilar, Ingrid K. Torsvik, Maria Joao Gil-da-Costa, Ella Kumirova, Ofelia Cruz-Martinez, Stefan Holm, Simon Bailey, Tim Hayden, Ulrich W. Thomale, Geert O. R. Janssens, Christof M. Kramm, Dannis G. van Vuurden

**Affiliations:** 1 Pediatric Oncology, Emma Children’s Hospital, Amsterdam UMC, Vrije Universiteit Amsterdam, Amsterdam, The Netherlands; 2 Princess Máxima Center for Pediatric Oncology, Utrecht, The Netherlands; 3 Department of Pediatric Hematology, Oncology and Stem Cell Transplantation, St Joan de Déu Children´s Hospital, Barcelona, Spain; 4 Department of Clinical Pharmacology & Pharmacy, Amsterdam UMC, Vrije Universiteit Amsterdam, Amsterdam, The Netherlands; 5 Department of Radiology & Nuclear Medicine, Amsterdam UMC, Vrije Universiteit Amsterdam, Amsterdam, The Netherlands; 6 Pediatric Oncology-Hematology Clinic, Archbishop Makarios III Hospital, Nicosia, Cyprus; 7 Department of Pediatric Hematology and Oncology, 2nd Faculty of Medicine, University Hospital Motol, Charles University, Prague, Czech Republic; 8 Pediatrics and Adolescent Medicine, University Hospital Rigshospitalet, Copenhagen, Denmark; 9 Department of Hematology and Oncology, Tallinn Children’s Hospital, Tallinn, Estonia; 10 Division of Hematology-Oncology and Stem Cell Transplantation, Children’s Hospital, Helsinki University Hospital, Helsinki, Finland; 11 Pediatric Hematology-Oncology, The Children’s Hospital, Reykjavík, Iceland; 12 Department of Pediatric Oncology and Hematology, Hospital of Lithuanian University of Health Sciences Kaunas Clinic, Kaunas, Lithuania; 13 Mater Dei Hospital, Valletta, Malta; 14 Children’s Memorial Health Institute, Warsaw, Poland; 15 Department of Pediatric Oncology, Oncology Institute Professor Doctor Alexandru Trestioreanu, Bucharest, Romania; 16 Department of Pediatric Oncology/Hematology, Children University Hospital, Kosice, Slovakia; 17 Division of Hemato-Oncology, Department of Pediatrics, University Medical Center Ljubljana, Ljubljana, Slovenia; 18 Pediatric Oncology and Hematology, Department of Pediatrics, University Hospital of Geneva, Geneva, Switzerland; 19 Pediatric Hematology-Oncology, Cerrahpasa Medical Faculty & Oncology Institute, Istanbul University, Istanbul, Turkey; 20 Department of Pediatrics and Adolescent Medicine, Medical University of Vienna, Vienna, Austria; 21 Universitair Ziekenhuis Leuven, Leuven, Belgium; 22 Department of Oncology and Hematology, Children’s Hospital Zagreb, Zagreb, Croatia; 23 CHRU Hautepierre Strasbourg, Service de Pédiatrie Onco-Hématologie, Strasbourg, France; 24 Département de Cancérologie de l’enfant et de l’adoloscent, CLCC Institut Gustave Roussy, Villejuif, France; 25 First Department of Pediatrics, ‘Aghia Sofia’ Children’s Hospital, National and Kapodistrian University of Athens, Athens, Greece; 26 Second Department of Pediatrics, Semmelweis University, Budapest, Hungary; 27 Department of Pediatric Oncology, Our Lady’s Children’s Hospital, Crumlin, Dublin, Ireland; 28 Pediatric Oncology Unit, Fondazione IRCCS Istituto Nazionale dei Tumori, Milan, Italy; 29 Hospital de Pediatría, Centro Médico National Siglo XXI, Instituto Mexicano del Seguro Social, Jefatura de Servicio de Oncologia, Distrito Federal, Mexico; 30 Division of Oncology/Hematology, Department of Pediatrics, Haukeland University Hospital, Mons, Norway; 31 Pediatric Hematology and Oncology Division, University Hospital S. João Alameda Hernani Monteiro, Porto, Portugal; 32 Department of Neurooncology, Federal Research and Clinical Centre of Pediatric Hematology, Oncology and Immunology (FRC-PHOI), Moscow, Russia; 33 Department of Pediatric Hematology and Oncology, Department of Woman and Child Health, Karolinska University Hospital, Stockholm, Sweden; 34 Great North Children’s Hospital, Victoria Wing, Royal Victoria Infirmary, Newcastle upon Tyne, UK; 35 The DIPG Collaborative, Cincinnati, USA; 36 Pediatric Neurosurgery, Charité University Medical Center Berlin, Berlin, Germany; 37 Department of Radiation Oncology, University Medical Center Utrecht, Utrecht, The Netherlands; 38 Division of Pediatric Hematology and Oncology, University Medical Center Geottingen, Göttingen, Germany; 39 Department of Pediatric Oncology/Hematology, Amsterdam UMC, location VUmc, De Boelelaan 1117, 1081 HV Amsterdam, The Netherlands

**Keywords:** Diffuse intrinsic pontine glioma (DIPG), Diffuse midline glioma H3-K27 mutant (DMG K3-27M), Chemotherapy, Radiotherapy

## Abstract

**Introduction:**

Diffuse intrinsic pontine glioma (DIPG) is a rare clinically, neuro-radiologically, and molecularly defined malignancy of the brainstem with a median overall survival of approximately 11 months. Our aim is to evaluate the current tendency for its treatment in Europe in order to develop (inter)national consensus guidelines.

**Methods:**

Healthcare professionals specialized in DIPG were asked to fill in an online survey with questions regarding usual treatment strategies at diagnosis and at disease progression in their countries and/or their centers, respectively.

**Results:**

Seventy-four healthcare professionals responded to the survey, of which 87.8% were pediatric oncologists. Only 13.5% of the respondents biopsy all of their patients, 41.9% biopsy their patients infrequently. More than half of the respondents (54.1%) treated their patients with radiotherapy only at diagnosis, whereas 44.6% preferred radiotherapy combined with chemotherapy. When the disease progresses, treatment strategies became even more diverse, and the tendency for no treatment increased from 1.4% at diagnosis to 77.0% after second progression. 36.5% of the healthcare professionals treat children younger than 3 years differently than older children at diagnosis. This percentage decreased, when the disease progresses. Most of the participants (51.4%) included less than 25% of their patients in clinical trials.

**Conclusion:**

This survey demonstrates a large heterogeneity of treatment regimens, especially at disease progression. We emphasize the need for international consensus guidelines for the treatment of DIPG, possible by more collaborative clinical trials.

**Electronic supplementary material:**

The online version of this article (10.1007/s11060-019-03287-9) contains supplementary material, which is available to authorized users.

## Introduction

Children suffering from diffuse intrinsic pontine glioma (DIPG) face a dismal prognosis with a median overall survival of approximately 11 months, and a 2 year survival rate of 10% [1, 2]. Due to the delicate location of the tumor, surgical resection is not possible. To date, radiotherapy remains standard of care at diagnosis and confers a survival benefit of approximately 3 months [3]. Chemotherapy has not proven to be effective thus far [2].

In the recently revised World Health Organization (WHO) classification of central nervous system (CNS) tumors, the majority of DIPG has neuropathologically been reclassified within a novel tumor entity: diffuse midline glioma, H3-K27 mutant (DMG H3-K27M). This entity is defined as an infiltrative high-grade glioma, located in the brain midline, i.e. usually brainstem, spinal cord, cerebellum or thalamus, with astrocytic differentiation and K27M mutation in either H3F3A or HIST1H3B/C [4]. Up to 85% of the DIPGs harbor this mutation [5, 6]. Wild-type H3-K27 DIPGs have not yet been separately classified within the revised WHO classification, but show similar survival as H3-K27M DIPGs [1, 7].

Because of the poor prognosis and limited effect of current treatment strategies, there are no (inter)national guidelines for both radio- and chemotherapy, resulting in a heterogeneous application of treatment schedules, as shown by a Dutch national inventory [8]. In this European retrospective study, we aim to evaluate the current tendency for treatment (i.e. the applied type of treatments per country, including enrollment in clinical trials) in a purpose to (i) structure optional inclusion in collaborative clinical trials, and (ii) stimulate the development of (inter)national consensus guidelines for the approach of DIPG patients.

## Methods

An online survey was developed and distributed among healthcare professionals specialized in DIPG via the electronic mailing list of the International Society of Pediatric Oncology Europe Brain Tumor Group (SIOPE BTG; n = 414). The survey was published online via ThesisTools pro (www.thesistoolspro.com) and was open for participation for a total period of 10 weeks, from June 2018 to August 2018. Three weeks after the initial distribution the first reminder was sent, and after 6 weeks the second one.

The survey queried treatment regimens used in DIPG patients at diagnosis and at (first and subsequent) disease progression: initial treatment after diagnosis, second line treatment after first progression, and third line treatment after second progression. Participants were asked to describe the applied radiotherapy dose and fractionation, drugs, doses, and schedules, and whether patients participated in clinical trials or not. The full survey can be found in the supplementary material.

The data obtained from the survey were analyzed by descriptive statistical methods using IBM SPSS Statistics version 22.

## Results

Seventy-four health care professionals treating DIPG patients in Europe contributed to the online survey. Since this online survey was distributed via the electronic mailing list of the SIOPE BTG (n = 414), also including professionals not (directly) involved in the treatment of DIPG patients, and members of the SIOPE BTG group were asked to forward the survey to their colleagues outside the network who are also treating DIPG patients, it was not possible to determine the exact overall response rate.

Among the healthcare professionals who responded to the survey, 87.8% were pediatric oncologists. Others were radiation oncologists, pediatric neurosurgeons and child neurologists (Table 1). Most of the respondents (87.8%) treat up to 5 patients per year (range 1–13 patients, with one outlier of 35 patients in Russia). Table 1 contains demographic data of the respondents.

**Table 1:** Demographic data of the healthcare professionals treating DIPG patients who contributed to the online survey

	n = 74 (%)
**Function/specialty**	
Pediatric oncologist	65 (87.8)
Radiation oncologist	1 (1.4)
Pediatric neurosurgeon	5 (6.8)
Child neurologist	3 (4.1)
**DIPG national coordinator** ^a^	15 (20.3)
**DIPG trial coordinator**	13 (17.6)
**Number of DIPG patients treated per year**	
0–5	65 (87.8)
6–10	7 (9.5)
11–15	1 (1.4)
> 15	1 (1.4)
**Country**	
Netherlands	8 (10.8)
Germany	4 (5.4)
Spain	11 (14.9)
Italy	5 (6.8)
Belgium	5 (6.8)
France	8 (10.8)
United Kingdom	11 (14.9)
Switzerland	3 (4.1)
Sweden	1 (1.4)
Norway	2 (2.7)
Portugal	1 (1.4)
Russia	2 (2.7)
Slovakia	2 (2.7)
Slovenia	1 (1.4)
Lithuania	1 (1.4)
Hungary	1 (1.4)
Greece	2 (2.7)
Croatia	1 (1.4)
Czech Republic	1 (1.4)
Denmark	1 (1.4)
Austria	2 (2.7)
Australia	1 (1.4)

^a^Designated person per country who collects information about all DIPG patients for the European DIPG registry

### Biopsy and neuropathological diagnosis

Most of the healthcare professionals (41.9%) stated to biopsy their patients infrequently. Only 13.5% biopsy all DIPG patients, whereas 16.2% never biopsy DIPG patients (Table 2). 13.5% of the healthcare professionals described to have difficulties in their daily routine with the new neuropathological diagnosis of DMG H3-K27M. A more detailed explanation of the experienced difficulties is the fact that biopsies are required to determine the neuropathological diagnosis, although explaining the potential relevance and benefit of a biopsy to patients and their families is particularly difficult as the result-in most cases-will not influence treatment decisions. 68.9% of the respondents stated to treat non-pontine DMG H3-K27M like they treat DIPG (i.e. radiotherapy only or radiotherapy combined with chemotherapy) versus 31.1% who treat them like they treat HGG (i.e. with radiotherapy combined with chemotherapy, mainly temozolomide).

**Table 2:** Frequency of biopsy in DIPG patients

	n = 74 (%)
All patients	10 (13.5)
Most patients	21 (28.4)
Few patients	31 (41.9)
Never	12 (16.2)

### Treatment strategies

54.1% of the healthcare professionals considered radiotherapy as monotherapy to be standard of care at diagnosis (Table 3). Of these, 47.5% use conventionally-fractionated radiotherapy (54–60 Gy in 1.8–2.0 Gy fractions) versus 17.5% who prescribe a biologically equivalent hypo-fractionated dose of 30–40 Gy in 3.0–4.0 Gy fractions. 35% of respondents indicated that they give both conventionally- and hypo-fractionated radiotherapy to their DIPG patients. 44.6% of the respondents considered radiotherapy combined with chemotherapy standard of care at diagnosis, of which 39.4% combine radiotherapy with daily temozolomide, and 60.6% use other concomitant chemotherapy. Chemotherapy was only given together with a conventionally fractionated radiotherapy scheme.

**Table 3:** Provided first line therapy to DIPG patients by responding healthcare professionals

	n = 74 (%)
**Radiotherapy only**	**40 (54.1)**
CF RTx	19 (47.5)
HF RTx	7 (17.5)
CF RTx or HF RTx	14 (35.0)
**Radiotherapy combined with chemotherapy**	**33 (44.6)**
CF RTx + temozolomide	13 (39.4)
CF RTx + nimotuzumab + vinorelbine	6 (18.2)
CF RTx + other chemotherapeutics	14 (42.4)
**No treatment**	**1 (1.4)**

*CF RTx* Conventionally fractionated radiotherapy, 54–60 Gy in 1.8–2.0 Gy fractions, *HF RTx* Hypo-fractionated radiotherapy, 30–40 Gy in 3.0–4.0 Gy fractions

At first progression, 77.0% of the healthcare professionals considered re-irradiation to be standard of second-line care; 70.2% of these use re-irradiation only, and 29.8% combine re-irradiation with chemotherapy. Others described to use chemotherapy only, immunotherapy, or no treatment at all. At second progression (third line therapy), 77.0% of the respondents did not actively treat their patients and provided only palliative symptom control. Others stated to use several treatment strategies (Table 4).

**Table 4:** Provided second and third line therapy to DIPG patients (after first and second progression) by responding healthcare professionals

	n = 74 (%)
**Second line therapy**	
HF radiotherapy only	40 (54.1)
HF radiotherapy + chemotherapy	17 (23.0)
Chemotherapy only	6 (8.1)
Immunotherapy	2 (2.7)
No treatment	7 (9.5)
Other	2 (2.7)
**Third line therapy**	
HF Radiotherapy^a^ only	8 (10.8)
Chemotherapy only	5 (6.8)
HF Radiotherapy^a^ + chemotherapy	3 (4.1)
Immunotherapy	1 (1.4)
No treatment	57 (77.0)

*HF radiotherapy* hypo-fractionated radiotherapy, 30–40 Gy in 3.0-4.0 Gy fractions)

^a^Only when not provided as second line therapy

36.5% of the respondents stated to treat children younger than 3 years at diagnosis different than older children; starting with no treatment or chemotherapy only, instead of radiotherapy. The percentage of caregivers giving a different treatment decreased when the disease progressed, to 23.0% after first and 13.5% after second progression, respectively.

### Participation in clinical trials

Seventy-three percent of the healthcare professionals reported to have ongoing clinical trials in their hospital or another referable center in their country. 51.4% of the respondents stated that less than 25% of their patients participate in ongoing clinical trials (Table 5).

**Table 5:** Number of healthcare professionals who include patients in clinical trials

	n = 74 (%)
**Patients participating in clinical trials**	
< 25%	38 (51.4)
25–50%	8 (10.8)
50–75%	6 (8.1)
> 75%	22 (29.7)
**No clinical trials in center or nearby**	20 (27.0)

## Discussion

Regular exposure to larger numbers of DIPG patients remains low; up to 90% of the healthcare professionals treat less than five DIPG patients per year. This study confirms the need for more (inter)national collaboration to increase knowledge about the disease and reasonable treatment strategies.

Over the past 20 years, neuropathological diagnosis of tumors arising in the pons has been subject to change. In the late 90s, performing biopsies in children suffering from DIPG was not recommended because of the presumed risk of the biopsy procedures, and because of the fact that histological grading did not alter therapy outcome [9, 10]. This paradigm slowly shifted when reports showing the safety of biopsies were published [11]. In 2011, a multi-disciplinary consensus statement was published justifying biopsies as part of clinical trials to investigate tumor grading and biological markers important for treatment selection [12]. In 2015, German colleagues performed a smaller scale (n = 18) email survey, similar to ours, also within the SIOPE DIPG network, which demonstrated that at that time approximately 10% of the healthcare professionals biopsied their patients (prof. C.M. Kramm, unpublished data). The results of our study show an increase; currently 42% of the healthcare professionals state to biopsy all or most of their patients, compared to 16% who never biopsy their patients. This paradigm shift towards integrating biopsies in the work-up of DIPG patients could be explained by the increase of clinical trials over the years. Trials such as BIOMEDE with treatment decisions according to the molecular findings within performed biopsies, could be the main drivers behind this trend. Especially since our results show that healthcare professionals are reluctant to have an invasive procedure like a biopsy performed in DIPG patients, when it does not provide a benefit or therapeutic consequence.

This international survey study demonstrates that almost all DIPG patients are treated with radiotherapy at diagnosis, either alone (54.1%) or combined with chemotherapy (44.6%). In a Dutch cohort study covering 1990–2010, 79.6% of the DIPG patients were treated with radiotherapy at diagnosis, of whom 66% received radiotherapy alone, and 13.6% received radiotherapy combined with chemotherapy [8]. The observed modest shift towards combining radiotherapy with chemotherapy in our current study might, like the tendency to biopsy, be explained by the increased of the number of available clinical trials over the past decade.

Of the radiotherapy schedules, most healthcare professionals apply conventionally-fractionated radiotherapy at diagnosis, especially when patients participate in clinical trials. Our study shows that outside clinical trials some healthcare professionals tend to use hypo-fractionated radiotherapy because of its lower-burden regimen and as conventionally- or hypo-fractionated radiotherapy schedules in DIPG have been shown to result in equal overall survival rates [13].

Heterogeneity in treatment is demonstrated, especially when the disease progresses. Among all treatments given, re-irradiation (either alone or combined with chemotherapy) is mostly used after first progression, and has been proven to give a significant survival benefit of 3.4 months in DIPG patients [14]. At further progressions, treatment strategies such as chemotherapy alone or immunotherapy are also used. However, these numbers are low and are especially in case of chemotherapy mostly related to participation in clinical trials. After second progression, 77% of the healthcare professionals do not actively treat their patients, which is in line with previously published work within one country by Veldhuijzen van Zanten et al. [15]. Healthcare professionals prefer to provide palliative and end-of-life care in this last stage of the disease.

Looking at age-related differences in treatment, our study shows that 74.5% of the healthcare professionals treat children younger than three years the same as older children, whereas previous studies have shown that younger age at diagnosis is correlated to longer survival of DIPG patients [2, 16]. A possible explanation for this attitude, despite the significantly better prognosis of infant DIPG patients, might be because infant DIPGs are extremely rare and, therefore, the potentially better prognosis which can often be reached with chemotherapy alone, might not be general knowledge and/or accepted yet. The percentage of healthcare professionals who treat younger children different than older children decreases when the disease progresses because with progression the possible advantage of infant DIPG patients with regards to a potential better prognosis has obviously vanished. This is in line with the transition from active treatment to palliative and end-of-life care, which is less age dependent.

As mentioned before, the increased availability of clinical trials and increased knowledge on the biological background of DIPG over the past decades could explain the actual trend towards biopsies and the different treatment combinations (e.g. radiotherapy combined with chemotherapy). Our study, however, demonstrates that participation in clinical trials still remains low; more than half of the healthcare professionals include less than 25% of their patients in clinical trials. This is in agreement with the results of a previous study by van der Geest et al., who investigated participation in clinical trials of children with incurable cancers. They demonstrated that less than one-third of the patients participate in a clinical trial [17], which might be explained by (i) the ethical dilemma’s health care professionals face regarding mandatory parts of the trials, i.e. biopsies with no direct benefit for the patient; (ii) the travel distance to a study center, resulting in parents declining participation in the trial; and (iii) cultural differences. To illustrate, in some countries, parents/patients do not want to do anything but palliative care to minimize a child’s suffering, while in other countries parents/patients are willing to do everything to get any form of treatment. The latter is exemplified by the increased crowdfunding events over the past years, where parents raise money to participate in clinical trials abroad. This will add to the responsibilities of healthcare professionals, who have to help parents balancing between the benefits of participation in clinical trials and preserving the child’s needs and protect them from unnecessary treatment burden. For this purpose, we advocate more collaboration between countries, with the aim to bring clinical trials to the patients, instead of the patients to the trials. To do so, international disease networks and registries, such as the SIOPE DIPG network and the SIOPE DIPG Registry, could be the foundation of collaborative trials [18].

Our study again confirms the heterogeneity of the treatment of DIPG, on a European level. To date, there are still no guidelines for both chemo- and radiotherapy, resulting in a wide variety of treatment schedules, especially at disease progression. This research therefore calls for international (SIOPE) guidelines and/or treatment recommendations with patient outcome data registration via the DIOPE DIPG Registry. Putting our data in perspective of the past twenty years, our results show less heterogeneity over time; fewer treatment strategies are considered at diagnosis and progression. More collaboration on a European level and working together on clinical trials is recommended to combine international expertise and to work towards collaborative clinical trials with higher patient numbers and, hopefully, to achieve quicker results. This cooperation is the basis for trying to change the dismal prognosis of patients suffering from DIPG.

## Electronic supplementary material

Below is the link to the electronic supplementary material.

Supplementary material 1 (PDF 376 kb)
