# Supplementary material for: Diagnostics and treatment of diffuse intrinsic pontine glioma: where do we stand?
Source: J Neurooncol. 2019 Sep 14;145(1):177–84. doi: 10.1007/s11060-019-03287-9 (PMC6775536; doi:10.1007/s11060-019-03287-9)
Supplement: Supplementary file 1 — Supplementary material 1 (PDF 376 kb) [file 11060_2019_3287_MOESM1_ESM.pdf]

Diagnostics and treatment of diffuse intrinsic pontine glioma: Where do we stand?

Journal of Neuro-Oncology

Fatma E. El-Khouly [1,2], Sophie E.M. Veldhuijzen van Zanten [1,2], Vicente Santa-Maria Lopez [3], N. Harry Hendrikse [4,5], Gertjan J.L. Kaspers [1,2] *et al.*

[1] Emma Children's Hospital, Amsterdam UMC, Vrije Universiteit Amsterdam, Pediatric Oncology, Amsterdam, The Netherlands.

[2] Princess Máxima Center for Pediatric Oncology, Utrecht, The Netherlands

[3] St Joan de Déu Children´s Hospital, Department of Pediatric Hematology, Oncology and Stem Cell Transplantation, Barcelona, Spain

[4] Amsterdam UMC, Vrije Universiteit Amsterdam, Department of Clinical Pharmacology & Pharmacy, Amsterdam, The Netherlands

[5] Amsterdam UMC, Vrije Universiteit Amsterdam, Department of Radiology & Nuclear Medicine, Amsterdam, The Netherlands

CORRESPONDING AUTHOR

Fatma E. El-Khouly

Amsterdam UMC, location VUmc - Department of Pediatric Oncology/Hematology

De Boelelaan 1117

1081 HV Amsterdam

The Netherlands

Tel.: +31 20 4445056

Email: f.el-khouly@amsterdamumc.nl

Dear colleague,

In the SIOPE DIPG Network of the HGG/DIPG working group of SIOP Europe, we have set up a survey to obtain more detailed information about the treatment regimens of DIPG in- and outside Europe. With this data we aim to write a paper about the homo/heterogeneity of treatment of DIPG in Europe and beyond. We would like to ask you to fill in a short survey (+/- 10 min) about the treatment options you have in your institution, as detailed as possible.

Thank you in advance.

Kind regards, also on behalf of Dannis van Vuurden and Vicente Santa-Maria Lopez,

Fatma El-Khouly

Start survey

1. **Background information participant**

Name ____________

Country ____________

City ____________

Hospital ____________

Function/specialty ____________

DIPG National coordinator? (Yes/No) ____________

DIPG trial coordinator? (Yes/No) ____________

2. **How many DIPG patients do you treat per year or (in case of national/trial coordinator) do you oversee per year?**

____

3. **How many of these DIPG patients participate in ongoing clinical trials?**

- A < 25%
- B 25-50%
- C 50-75%
- D > 75%

4. Which clinical trials are currently ongoing in your country? (please mention trial name and hospital)

5. Are DIPG patients biopsied in your hospital?

- A Yes, all patients are biopsied
- B Most patients are biopsied
- C Patientst are biopsied infrequently
- D No, we never perfom a biopsy on DIPG patients

6. Is it confusing for your daily clicnial routine that there is now a radiological diagnosis of DIPG and a neuropathological diagnosis of a diffuse midline glioma WHO IV, H3K27M mutant? If yes, could you briefly explain these difficulties/confusions?

- A Yes
- B No

7. Do you treat non-pontine diffuse midline gliomas WHO IV, H3K27M mutant, like DIPG? If no, could you briefly explain how you treat these patients?

- A Yes
- B No

Next - First line therapy

First line therapy

The following questions are about initial treatment, direclty after diagnosis.

8. When a patient is diagnosed with DIPG, what do you consider to be the standard first line treatment? Please mention (generic) name, dose, frequency and treatment duration.

- A Radiotherapy only
- B Chemotherapy only
- C Radiotherapy + chemotherapy
- D Immunotherapy
- E No treatment
- F Other

9. There seems to be a difference between younger (≤ 3 years) and older (> 3 years) children suffering from DIPG. Younger children seem to have a better prognosis. Do you treat these younger children different than older children at diagnosis?

- A Yes
- B No

10. If yes, please describe what you do differently with these children.

11. Other comments regarding first line therapy

Next - Second line therapy

Second line therapy

The following questions are about the treatment you offer after first progression.

12. When a patient has progressive disease after initial therapy, what do you consider to be the most appropriate second line treatment? Please mention (generic) name, dose, frequency and treatment duration.

- A Radiotherapy only
- B Chemotherapy only
- C Radiotherapy + chemotherapy
- D Immunotherapy
- E No treatment
- F Other

13. Do you treat younger children (≤ 3 years) different than older children (> 3 years) after first progression?

- A Yes
- B No

14. If yes, please describe what you do differently with these children.

15. Other comments regarding second line therapy (after first progression).

Next - Third line therapy

Third line therapy

The following questions are about the treatment you offer after second progression.

16. When a patient has progressive disease for the second time, what do you consider to be the most appropriate third line treatment? Please mention (generic) name, dose, frequency and treatment duration.

- A Radiotherapy only
- B Chemotherapy only
- C Radiotherapy + chemotherapy
- D Immunotherapy
- E No treatment
- F Other

17. Do you treat younger children (≤ 3 years) different than older children (> 3 years) after second progression?

- A Yes
- B No

18. If yes, please describe what you do differently with these children.

19. Other comments regarding third line therapy (after second progression).

Finish and save survey

Thank you for your contribution to this survey. After analyzing the data, we will inform you about the results.
